# Scoping Review of Letrozole in Assisted Reproductive Cycles: Efficacy and Outcomes Across Infertility Causes

**DOI:** 10.3390/healthcare13131486

**Published:** 2025-06-20

**Authors:** Anastasios Potiris, Efthalia Moustakli, Fani Migka, Athanasios Zikopoulos, Chrysi Christodoulaki, Argyro Papadopoulou, Nikolaos Kathopoulis, Charalampos Theofanakis, Periklis Panagopoulos, Ekaterini Domali, Peter Drakakis, Sofoklis Stavros

**Affiliations:** 1 Third Department of Obstetrics and Gynecology, University General Hospital “ATTIKON”, Medical School, National and Kapodistrian University of Athens, 12462 Athens, Greece; apotiris@med.uoa.gr (A.P.); thanzik92@gmail.com (A.Z.); christodoulakichr@hotmail.com (C.C.); ch.theofanakis@gmail.com (C.T.); perpanag@med.uoa.gr (P.P.); pdrakakis@med.uoa.gr (P.D.); 2 Laboratory of Medical Genetics, Faculty of Medicine, School of Health Sciences, University of Ioannina, 45110 Ioannina, Greece; thaleia.moustakli@gmail.com; 3 First Department of Obstetrics and Gynecology, Alexandra Hospital, Medical School, National and Kapodistrian University of Athens, 11528 Athens, Greece; fanimgk@med.uoa.gr (F.M.); argypapadopoulou@gmail.com (A.P.); nickatho@gmail.com (N.K.); kdomali@yahoo.fr (E.D.)

**Keywords:** letrozole, infertility, assisted reproductive techniques (ART), ovulation induction, aromatase inhibitors

## Abstract

**Background:** Infertility affects 8–12% of couples globally, with causes including hormonal and structural abnormalities. Letrozole, an aromatase inhibitor, is commonly used for ovulation induction, but its role in various assisted reproductive technologies (ARTs) and across different subgroups of infertile women remains unclear. **Objective:** This scoping review aimed to map the existing evidence on the use of letrozole in assisted reproductive cycles, focusing on reproductive outcomes and its application across different patient populations. **Methods:** A scoping review was conducted following the PRISMA-ScR guidelines. Twelve studies—including randomized controlled trials and retrospective cohorts—were identified through a structured search strategy. Studies comparing letrozole alone or in combination with gonadotropins/clomiphene to other stimulation protocols were included. Data were charted across multiple outcomes including oocyte yield, implantation, pregnancy, miscarriage, and live birth rates. **Results:** Evidence suggests that letrozole-based protocols may enhance oocyte yield and improve reproductive outcomes in certain settings. The highest implantation rate (57%) was observed in natural cycles, while the letrozole-only group showed the highest clinical pregnancy (50.57%) and live birth rates (45.58%). Combination protocols achieved the highest ongoing pregnancy rate (58.3%), with the lowest miscarriage rate (14.86%) in the letrozole-only group. **Conclusions:** Letrozole appears to be a versatile agent in ART, especially for patients requiring reduced gonadotropin doses or estradiol modulation. This scoping review highlights the need for further research to clarify its optimal use across different infertility subgroups and ART modalities.

## 1. Introduction

Infertility is a significant public health issue, affecting approximately 8–12% of couples worldwide [1]. According to the World Health Organization (WHO), infertility is defined as the inability to achieve pregnancy after at least one year of regular, unprotected sexual intercourse. It is classified as primary when no prior pregnancy has been achieved and secondary when there is a history of previous pregnancies, regardless of their outcome [2].

Both female and male factors contribute to infertility, with genetic, hormonal, and environmental influences playing critical roles [1,2]. Female infertility is multifaceted and often results from ovulation disorders such as polycystic ovary syndrome (PCOS), endometriosis, premature ovarian insufficiency, Turner syndrome, and hyperprolactinemia [3,4]. Male infertility, which accounts for approximately 20–30% of cases, is primarily associated with poor semen quality, low sperm motility (asthenozoospermia), abnormal sperm morphology, and low sperm count (oligospermia) [1,5,6]. Additionally, immunological disorders or chromosomal abnormalities in either partner can impair the ability to conceive and maintain a healthy pregnancy [2,3,7]. Addressing these underlying conditions is crucial for restoring fertility.

The management of infertility typically involves three main approaches: pharmaceutical therapy, surgical interventions (primarily endoscopy), and assisted reproductive technology (ART) [2]. ART has advanced significantly over the past decades, with in vitro fertilization (IVF) and intracytoplasmic sperm injection (ICSI) being the most commonly used techniques [3,8,9]. Modern ART methods have led to higher pregnancy and live birth rates, particularly in patients with complex infertility causes, such as PCOS, diminished ovarian reserve, and breast cancer [2,3,4].

The basis of the most common ART indications is biochemical and metabolic imbalance. For example, insulin resistance, hyperinsulinemia, and hyperandrogenism are hallmarks of PCOS, a major cause of anovulatory infertility. These conditions interfere with follicular development and disturb the hypothalamic–pituitary–ovarian (HPO) axis. Prolonged inflammation and elevated local estrogen production are two key pathophysiological features of endometriosis that reduce endometrial receptivity and hinder successful embryo implantation [10,11]. Diminished ovarian reserve and altered gonadotropin signaling are often linked to premature ovarian insufficiency. These changes could result from autoimmune diseases, genetic predispositions, or iatrogenic factors [12]. The requirement for systemic estrogen suppression to prevent cancer cell growth makes fertility preservation more challenging in hormone receptor-positive breast cancer. Letrozole helps regulate estrogen levels and restore ovulatory function in hormonal imbalances, necessitating a deep understanding of the underlying molecular mechanisms [13].

Beyond its role in reproductive medicine, letrozole is widely used for the treatment of hormone receptor-positive (HR+) breast cancer, particularly in postmenopausal women [8,14,15]. As estrogens promote the growth of HR+ cancer cells, letrozole effectively lowers estrogen levels by inhibiting aromatase and suppressing tumor progression. This mechanism also makes letrozole a valuable tool for fertility preservation in women with hormone-sensitive cancers, as it allows for controlled ovarian stimulation while minimizing estrogen exposure [8,15,16].

In the field of ART and ovulation induction, letrozole has emerged as a widely used and effective option. It is a third-generation, non-steroidal selective aromatase inhibitor that suppresses estrogen production, thereby increasing follicle-stimulating hormone (FSH) secretion and promoting follicular development [3,15,17]. Letrozole’s ability to avoid an excessive elevation of luteinizing hormone (LH) minimizes the risk of hyperandrogenism and hormonal imbalances, unlike clomiphene citrate [18,19]. Studies have demonstrated that letrozole improves fertilization outcomes while minimizing the risk of ovarian hyperstimulation syndrome (OHSS), a severe complication associated with gonadotropin-based fertility treatments. Compared to clomiphene citrate, letrozole has shown superior efficacy in ovulation induction, with fewer adverse effects such as endometrial thinning and high rates of multiple pregnancies [2,15].

This review aims to evaluate the role of letrozole in assisted reproductive cycles, analyzing its impact on reproductive outcomes and its effectiveness in specific patient subgroups undergoing ART.

## 2. Letrozole in Infertility and Assisted Reproductive Technology

Letrozole has emerged as a valuable tool in fertility preservation for women with hormone-sensitive cancers, allowing ovarian stimulation while minimizing estrogen exposure. It is often combined with low doses of gonadotropins to optimize oocyte yield and ensure safety [13,14].

In ovulation induction, letrozole is a first-line treatment, particularly for women with PCOS. By reducing estrogen levels, letrozole promotes an increase in FSH secretion, facilitating follicular development. Unlike clomiphene citrate, letrozole avoids excessive LH elevation, reducing the risk of hyperandrogenism and hormonal imbalance [18,19].

In ART protocols, letrozole is used for ovulation induction in anovulatory women or as an adjunct to enhance ovulation in ovulatory women. It is commonly applied in intrauterine insemination (IUI) cycles and IVF/ICSI protocols. Letrozole reduces the total gonadotropin dose required, improving ovarian response, particularly in poor responders [8,16].

In the context of fertility preservation, letrozole is particularly valuable for women who are undergoing treatments such as chemotherapy or radiation. Letrozole helps reduce estrogen exposure, which can be crucial for hormone-sensitive cancers. Promoting ovarian function without significantly raising estrogen levels facilitates oocyte collection for future use without compromising the patient’s fertility. This makes it an important tool for preserving fertility in women facing medical treatments that could otherwise lead to infertility [20].

Letrozole is also effective in frozen embryo transfer (FET) cycles, enhancing endometrial receptivity and increasing clinical pregnancy and live birth rates, while reducing miscarriage rates compared to natural or artificial cycles [21,22]. Additionally, its ability to mitigate OHSS further underscores its safety and utility in ART protocols [14,19].

## 3. Methods

### 3.1. Study Design

This scoping review was conducted in accordance with the PRISMA-ScR (Preferred Reporting Items for Systematic Reviews and Meta-Analyses extension for Scoping Reviews) guidelines. The PRISMA checklist for scoping reviews is provided as Supplementary Material (Table S1). The objective was to systematically map the existing evidence on the use of letrozole in assisted reproductive technologies (ART) and identify key concepts, knowledge gaps, and patterns across different infertility subgroups. After removing duplicates (*n* = 918), the titles and abstracts of 1478 studies were screened for relevance. Of these, 1401 studies were excluded for not meeting the inclusion criteria. From the remaining 77 studies, 64 were excluded due to insufficient data or irrelevance to the research question. One study was later retracted due to a lack of data, leaving a final selection of 12 studies (RCTs and retrospective cohort studies) for detailed analysis and comparison with international literature. The search was conducted until February 2024.

### 3.2. Search Strategy

A comprehensive literature search was performed across PubMed, MEDLINE, and Scopus for studies published between January 2014 and February 2024. The search strategy included combinations of keywords and MeSH terms such as “letrozole”, “infertility”, “assisted reproductive technology”, “IVF”, and “ovulation induction” using Boolean operators (e.g., “letrozole AND IVF”, “letrozole OR clomiphene”).

**Figure 1:**
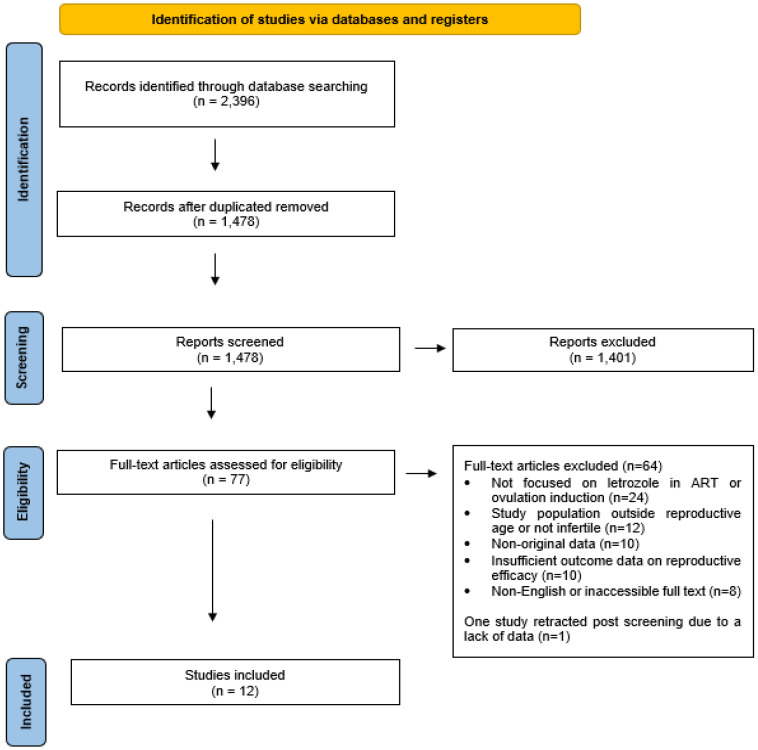
PRISMA flow diagram for the scoping review process.

The initial search yielded 2396 results. After removing duplicates (*n* = 918), 1478 titles and abstracts were screened. Following eligibility assessment, 12 studies (including randomized controlled trials and retrospective cohort studies) met the inclusion criteria for final analysis. Figure 1 depicts the study selection process.

### 3.3. Eligibility Criteria, Study Selection and Data Charting

This scoping review included studies that were peer-reviewed, published in English between January 2014 and February 2024, and evaluated the use of letrozole in assisted reproductive technology (ART). Studies were eligible if they involved infertile women undergoing procedures such as in vitro fertilization (IVF), intrauterine insemination (IUI), intracytoplasmic sperm injection (ICSI), frozen embryo transfer (FET), or fertility preservation, and reported on at least one reproductive outcome (e.g., oocyte yield, pregnancy, or live birth). Exclusion criteria included non-clinical studies, sample sizes below a defined threshold, studies lacking efficacy or safety outcomes, and participants with gynecologic surgeries, congenital uterine abnormalities, chronic or autoimmune diseases, acute illness, or age above 42 years.

Following the initial search, 2396 records were identified and 918 duplicates were removed. The remaining 1478 titles and abstracts were screened for relevance, leading to the exclusion of 1401 articles that did not meet inclusion criteria. Of the 77 full-text studies assessed for eligibility, 64 were excluded due to insufficient data or irrelevance to the research objective. One study was retracted post-screening, resulting in 12 studies being included for final analysis.

Two reviewers independently conducted the screening and selection process. Data from the included studies were charted using a standardized form capturing key characteristics such as study design, population demographics, intervention type and dosage, ART procedure, infertility diagnosis, and reported outcomes. This approach facilitated a comprehensive overview of how letrozole has been used across various patient subgroups and clinical settings.

### 3.4. Population and Subgroups

The selected studies examined a total of 17,623 infertile women who underwent ART. Participants were categorized based on underlying infertility diagnoses, including polycystic ovary syndrome (PCOS), endometriosis, poor ovarian response, gynecologic cancer (for fertility preservation), unexplained infertility, male factor infertility, and other causes (e.g., tubal factor or idiopathic infertility) (Table 1). Mean participant age was 33.03 years (SD = 3.79), and mean BMI was 23.22 kg/m^2^ (SD = 3.10), with slight variations between intervention groups (Table 2).

### 3.5. Interventions

Interventions across the included studies varied but were generally classified into five groups: (1) letrozole alone, administered at doses ranging from 2.5 mg to 7.5 mg daily for five days; (2) letrozole in combination with other agents such as gonadotropins, GnRH agonists, progesterone, or leuprolide; (3) non-letrozole regimens including clomiphene citrate, hormone replacement therapy, gonadotropins, or GnRH analogs; (4) natural cycles without pharmacological stimulation; and (5) control groups as defined by individual study protocols (Table 3). Dosage and treatment duration were adjusted based on patient profiles and specific study designs, with higher doses typically applied to poor responders or women with diminished ovarian reserve.

### 3.6. Outcomes of Interest

The primary outcomes extracted from the included studies focused on the effectiveness of letrozole in ART and included the number of oocytes retrieved, implantation rate, clinical pregnancy rate, ongoing pregnancy rate, miscarriage rate, and live birth rate. Secondary outcomes involved subgroup-specific responses to letrozole, with particular attention to populations such as women with PCOS, endometriosis, poor ovarian response, and those undergoing fertility preservation due to gynecologic cancer.

### 3.7. Definitions

For consistency across studies, the following standardized definitions were used: Oocyte yield refers to the total number of oocytes retrieved after controlled ovarian stimulation. Implantation rate is defined as the number of gestational sacs observed via ultrasound divided by the number of embryos transferred. Clinical pregnancy rate indicates the proportion of treatment cycles in which an intrauterine fetal heartbeat was confirmed by ultrasound. Ongoing pregnancy rate refers to pregnancies that progressed beyond the first trimester, typically beyond 10 gestational weeks. Miscarriage rate captures the percentage of clinical pregnancies that ended in pregnancy loss prior to 28 weeks of gestation. Live birth rate is defined as the proportion of treatment cycles resulting in a live birth and is considered the most definitive indicator of ART success. Of 77 studies, 64 were excluded due to insufficient data or irrelevance to the research question. Further, 1 study was later retracted due to a lack of data, leaving a final selection of 12 studies (RCTs and retrospective cohort studies) for detailed analysis and comparison with international literature. The search was conducted until February 2024.

## 4. Results

### 4.1. Primary Outcomes Analysis

A total of 17,623 infertile women undergoing assisted reproductive technology (ART) were analyzed across 12 studies, including both randomized controlled trials (RCTs) and retrospective cohort studies. The final selection included participants diagnosed with polycystic ovary syndrome (PCOS), diminished ovarian reserve, endometriosis, unexplained infertility, and gynecologic cancers.

Primary outcomes were categorized by infertility subgroups. Among women with PCOS, letrozole significantly improved oocyte retrieval rates and endometrial receptivity compared to clomiphene citrate (Table 4). In patients with endometriosis, letrozole resulted in a higher implantation rate (47.53%) compared to conventional protocols (25.8%), although this difference was not statistically significant. For cases of unexplained infertility, pregnancy rates were higher in letrozole-only protocols than in combination regimens. These findings suggest that the most substantial benefits of letrozole were observed in PCOS and unexplained infertility groups.

Implantation rate was reported in five studies. The highest average rate was observed in the letrozole-only group (47.53%), followed by the combination group (34.61%) and other regimens (25.8%) (Table 5). Although letrozole alone showed improved implantation outcomes, the difference was not statistically significant (*p* = 0.096).

The clinical pregnancy rate was documented in ten studies. Letrozole-only protocols had the highest average rate (50.57%), followed by combination regimens (41.46%) and other protocols (35.48%) (Table 6). Natural cycle protocols reported a rate of 42.4%. The differences were not statistically significant (*p* = 0.42).

Ongoing pregnancy rates were reported in four studies. The letrozole-only group had an average rate of 38.29%, compared to 58.3% in combination therapies and 25.98% in other regimens (Table 7). Again, no statistically significant difference was found (*p* = 0.86).

Miscarriage rates were reported in seven studies. Letrozole-only protocols had the lowest average miscarriage rate at 14.86%, compared to 16.89% in combination regimens and 16.8% in other treatments (Table 8). Natural cycles had the highest average rate (20.05%). Differences were not statistically significant (*p* = 0.84).

Live birth rates were reported in seven studies. Letrozole-only regimens showed the highest average live birth rate at 45.58%, followed by combination therapies (37.79%) and other regimens (36.98%) (Table 9). Natural cycles reported a rate of 33.25%. No statistically significant differences were found (*p* = 0.5).

### 4.2. Secondary Outcomes

Letrozole demonstrated several beneficial secondary outcomes beyond those already reported in the primary outcome analysis. In PCOS populations, for instance, letrozole was associated with reduced estrogen levels and a lower need for gonadotropin doses, thereby improving overall cycle efficiency when used alongside other therapies. Additionally, letrozole enhanced endometrial receptivity, suggesting the potential for improved reproductive outcomes in PCOS patients [8].

## 5. Discussion

Letrozole has become a common adjunct in ovarian stimulation protocols, particularly for women with conditions like PCOS, unexplained infertility, and endometriosis. Several studies have explored its potential to enhance ART outcomes, including oocyte retrieval, fertilization, and pregnancy rates. However, the existing literature varies significantly in patient populations and treatment protocols. Factors such as obesity—an important contributor to infertility through its effects on ovulation, hormonal balance, and endometrial receptivity—also influence ART outcomes but were inconsistently reported across studies. This variability complicates a general recommendation for the use of letrozole across all ART cases. To better understand where letrozole is most beneficial, the following sections will explore its effects in different patient groups, and its use in various ART protocols.

### 5.1. Effectiveness of Letrozole in Oocyte Retrieval

#### 5.1.1. PCOS Population

Letrozole improves oocyte retrieval in PCOS, with consistent evidence supporting its combined use with gonadotropins. It also helps minimize OHSS risk. Several studies, including a meta-analysis by Baradwan et al. (2024), demonstrate that when letrozole is combined with gonadotropins, it significantly increases the number of oocytes retrieved compared to gonadotropin-only regimens [32]. This combination regimen also reduces the total gonadotropin dose required, thus optimizing the treatment cost and patient convenience.

These findings are supported by studies like those from Bastu and colleagues (2016), who found that combining letrozole with gonadotropins did not significantly enhance oocyte yield or quality but did lower the required gonadotropin dose [25]. Additionally, research by Kaçar et al. (2022) observed that letrozole enhanced oocyte yield and improved fertilization rates in poor responders undergoing GnRH-antagonist protocols [33].

#### 5.1.2. Unexplained Infertility

In women with unexplained infertility, the response to ovarian stimulation can be more variable. Letrozole may modestly improve oocyte yield in unexplained infertility, though effects on implantation and pregnancy remain inconsistent [34].

For example, a study by Mojtahedi et al. (2023) found that when letrozole was added to stimulation protocols, it did not lead to significant improvements in implantation or clinical pregnancy rates in women with unexplained infertility [35]. Similarly, Wang et al. (2019) showed that the impact on implantation rates with letrozole was not statistically significant [31].

#### 5.1.3. Endometriosis and Poor Responders

In endometriosis and poor responders, letrozole has shown mixed results. Some studies report improved oocyte yield and fertilization rates, but evidence remains inconsistent and patient-dependent [33]. However, the impact of letrozole on oocyte retrieval in these populations is less consistent, and the results vary depending on the severity of endometriosis or the degree of ovarian reserve.

Similarly, research by Mojtahedi and Bayar found that letrozole did not significantly enhance oocyte retrieval or embryo quality for women with endometriosis and ovulatory infertility [35,36]. However, other studies, like those from Cantor et al. (2019), showed superior IVF outcomes when combining letrozole with leuprolide acetate for women with endometriosis [26].

### 5.2. Fertilization and Embryo Quality

#### 5.2.1. PCOS

Letrozole may improve fertilization in PCOS and unexplained infertility populations, although embryo quality benefits are variable. Its impact is more pronounced when addressing OHSS risk than embryo quality itself. However, while letrozole improves oocyte quantity, its effects on embryo quality remain mixed, and further studies are required to clarify this relationship [34].

A randomized clinical trial by Bastu and colleagues (2016) found no significant improvement in oocyte yield or quality with letrozole, but it did reduce the required gonadotropin dose [25].

#### 5.2.2. Unexplained Infertility and Other Populations

In women with unexplained infertility, the combination of letrozole and gonadotropins has shown promise in improving fertilization rates, but its effect on embryo quality has been less consistent. For women who do not have an underlying ovarian dysfunction like PCOS, the benefit of adding letrozole to stimulation protocols is less clear. Studies show that letrozole increases oocyte yield but does not always improve the subsequent fertilization or embryo quality to the same extent as in PCOS patients.

A meta-analysis by Zhang et al. (2022) comparing endometrial preparation methods for FET in PCOS patients supported these findings [30]. The study, analyzing four retrospective studies from Asia, concluded that implantation rates did not significantly differ between mild ovarian stimulation (including letrozole) and artificial hormone cycles [35].

### 5.3. Pregnancy and Live Birth Rates

#### 5.3.1. PCOS and Ovulatory Dysfunction

Clinical pregnancy rates are highest in PCOS populations using letrozole. Live birth rates are more variable, potentially reflecting protocol differences. In unexplained infertility and endometriosis, letrozole’s impact remains less predictable. A meta-analysis by Baradwan et al. (2024) demonstrated that letrozole-based regimens were superior to clomiphene citrate for improving pregnancy rates in women with PCOS [32]. Despite these findings, live birth rates remain mixed, with some studies reporting no significant improvement compared to other stimulation protocols. The variability in results likely reflects differences in protocol designs and patient characteristics.

In a cohort study, Zhang et al. (2022) found that letrozole-FET cycles had a significantly higher pregnancy rate compared to hormone replacement therapy cycles [30].

#### 5.3.2. Endometriosis and Poor Responders

In women with endometriosis or poor responders, letrozole’s effect on pregnancy rates is less significant. For example, studies by Mojtahedi et al. (2023) found no difference in pregnancy rates between letrozole and gonadotropin-only protocols in women with endometriosis [35]. While letrozole helps reduce gonadotropin dose, the benefit in terms of improving clinical pregnancy rates is not as evident. Poor responders may benefit from the gonadotropin-sparing effects of letrozole, but its impact on pregnancy rates varies depending on factors such as age and ovarian reserve.

### 5.4. Miscarriage Rate

Evidence on the impact of letrozole on miscarriage rates is inconclusive. Some studies suggest lower miscarriage rates in letrozole cycles, particularly among women with PCOS. Lou et al. (2022) reported a reduced incidence of early miscarriage, although this finding has not been consistently replicated across the literature [21]. More robust data are needed to draw definitive conclusions regarding this outcome.

### 5.5. Comparison with Existing Literature

The effectiveness of letrozole in ART varies depending on patient characteristics and treatment protocols. This research shows that letrozole improves oocyte retrieval and clinical pregnancy rates, especially when used in conjunction with gonadotropins, and offers substantial advantages, especially for women with PCOS. The superiority of letrozole-based regimens over clomiphene citrate in PCOS patients is also supported by Baradwan et al. (2024), consistent with these findings [32]. Furthermore, consistent with Kaçar et al. (2022), our review confirms that adding letrozole to gonadotropin protocols can optimize oocyte yield while reducing OHSS risk [33].

Across studies, letrozole doses ranged from 2.5 mg to 7.5 mg per day for five consecutive days. Lower doses (2.5–5 mg) were primarily used for ovulation induction in PCOS patients, while higher doses (7.5 mg) were often applied in ART cycles requiring greater follicular stimulation. However, no universal consensus exists on the optimal dosage, highlighting the need for further randomized controlled trials to refine dosing strategies for different patient populations [37].

However, results are less consistent for unexplained infertility, endometriosis, and poor responders [10]. In women with unexplained infertility, letrozole may enhance ovarian responsiveness; nevertheless, its effect on fertilization and pregnancy rates is still unclear. This is consistent with the findings of Mojtahedi et al. (2023), who showed no discernible benefit of letrozole over gonadotropin-only protocols for endometriosis patients’ pregnancy outcomes [35]. Additionally, our results are still equivocal, despite some studies, such as Lou et al., 2022 [21] suggesting that letrozole cycles may be linked to lower miscarriage rates.

Letrozole’s benefits are most consistent in PCOS, with evidence supporting improved oocyte yield and pregnancy outcomes. Its role in other populations is still under investigation. Further RCTs are needed to define optimal protocols across diverse infertility profiles [8].

## 6. Consideration of Statistical Significance

Several studies have demonstrated that letrozole improves outcomes like oocyte retrieval and lowers gonadotropin requirements. However, not all of these findings are statistically significant. This disparity underscores the importance of exercising caution when evaluating letrozole’s clinical advantages, especially concerning pregnancy and live birth rates. Larger and more rigorous trials are needed to properly evaluate its efficacy in ART cycles. These studies would help reduce methodological discrepancies and provide more robust data, which is essential for informed clinical decision making.

## 7. Limitations of the Study

There are several limitations to this review. The included studies consist of both retrospective studies and RCTs, which may limit generalizability and introduce bias. Variations in patient populations, such as differences in conditions like endometriosis and PCOS, can affect the outcomes. Additionally, differences in treatment plans, such as dosing regimens and medication combinations, complicate comparisons between studies. Many studies have focused on short-term outcomes, with limited data on long-term results, such as live birth rates. Furthermore, the potential for publication bias must also be considered.

Key clinical variables, such as metformin use and body mass index (BMI), were not consistently reported across the studies, limiting our ability to assess the impact of these factors on ART outcomes. Due to the lack of access to raw data and inconsistent outcome reporting, comparative statistical analyses (e.g., chi-square tests or logistic regression) could not be conducted.

While letrozole has shown promising results in certain ART protocols, the lack of large-scale, well-designed prospective randomized controlled trials limits the ability to draw definitive conclusions about its optimal use. Future research should prioritize standardizing dosage regimens, treatment durations, and patient selection criteria to improve reproducibility and clinical applicability.

## 8. Conclusions

The strength of this scoping review is that it synthesizes findings from twelve primary studies and additional international literature on letrozole’s role in assisted reproductive cycles. Additionally, a novelty of this review is that it focuses on certain patient groups that would potentially benefit the most from letrozole administration. Letrozole offers advantages such as increased oocyte retrieval and reduced gonadotropin use, though its impact on oocyte quality remains debated. Combining letrozole with other treatments may improve ovarian stimulation, but therapy should be individualized based on patient profiles and goals.

Implantation rates tend to be higher with letrozole compared to other protocols, although small sample sizes and varied methodologies limit broad generalization. Some studies show improved clinical and ongoing pregnancy rates, while others report no significant differences compared to natural cycles. Letrozole may also lower miscarriage rates, particularly compared to natural cycles and hormone replacement therapy, though further research is needed.

**Figure 2:**
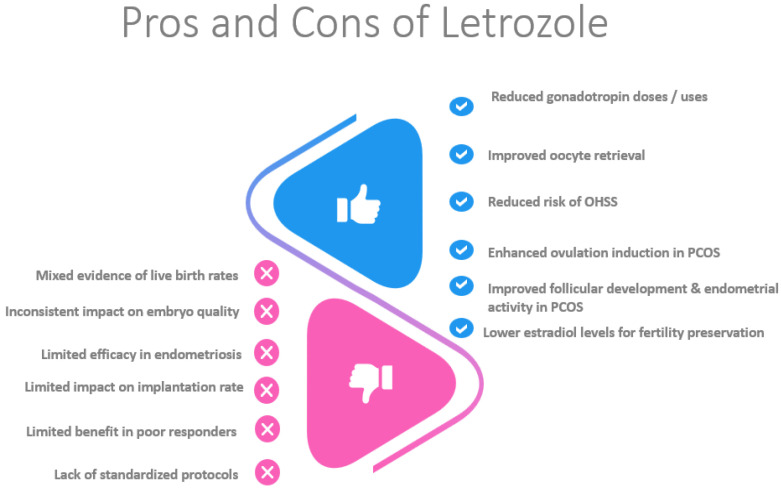
This figure summarizes the benefits and drawbacks of letrozole.

Letrozole provides specific benefits in certain patient groups. In women with PCOS, it improves cycle efficiency, reduces gonadotropin doses, and enhances follicular development. In women with endometriosis, letrozole reduces the risk of OHSS and improves live birth rates. For poor responders, letrozole shortens the stimulation duration and reduces gonadotropin use, maintaining comparable pregnancy rates. Additionally, it facilitates fertility preservation in women with hormone-sensitive cancers by lowering estradiol levels (Figure 2).

Despite these promising results, variability in study outcomes highlights the need for more large-scale, randomized controlled trials. Future research should clarify optimal dosing, protocol combinations, and long-term reproductive outcomes to refine letrozole’s role in reproductive medicine.

## Figures and Tables

**Table 1:** Participant distribution by infertility diagnosis. Summary of participants (n) categorized by infertility type across included studies: Polycystic Ovary Syndrome (PCOS), Endometriosis, Poor Ovarian Response, Gynecologic Cancer, Unexplained Infertility, Male Factor, and Other causes (including idiopathic, tubal factor, and mixed diagnoses).

Type of Intervention	Number of Participants (n)
Natural Cycle	6475
Letrozole	5676
Letrozole in Combination	653
Other Regimens	4732
Control Group	87
Total	17,623

**Table 2:** Mean age and BMI by intervention group. Age and BMI mean values and standard deviations (SD) for every intervention group. The BMI (in kg/m^2^) and mean age (in years) for natural cycle, letrozole, letrozole in combination, and other regimens are shown in the table along with the matching SD. Data not reported in a single study is indicated by values with an ‘a’, and data not reported in several studies is indicated by values with a ‘b’.

Intervention	Mean Age (SD) (Years)	Mean BMI (SD) (kg/m^2^)	*p*-Value
Natural Cycle	36.15 (2.45)	20.9 ^a^ (0.1 ^a^)	
Letrozole	31.91 (3.54)	22.21 (2.59)	
Letrozole in Combination	34.96 (3.79 ^a^)	23.45 ^a^ (4.05 ^b^)	
Other Regimes	31.49 (4.21 ^a^)	23.85 ^a^ (3.3 ^b^)	
Overall *p*-value			<0.0001

**Table 3:** Participants distribution by intervention type. The number of participants (n) is shown in the table according to the intervention group: natural cycle, letrozole alone, letrozole in combination (e.g., with gonadotropins, GnRH agonists, or other agents), other regimens (including clomiphene citrate, hormone replacement therapy, or gonadotropins alone), and control group.

Type of Infertility	Number of Participants (n)
Polycystic Ovary Syndrome	4260
Endometriosis	1142
Poor Responders	747
Gynecologic Cancer	341
Unexplained Infertility	3848
Male Factor	2595
Other (idiopathic, tubal factor, and others)	4249

**Table 4:** Summary of oocytes retrieved by treatment group. The mean number of oocytes retrieved per treatment group is presented below, with standard deviations (SD) in parentheses. Total sample sizes (n) are also included. The treatment groups include: letrozole alone (mean = 11), letrozole in combination (mean = 10.46), and other regimens (mean = 8.62). No statistically significant difference was observed among groups (*p* = 0.915). Data from Sun et al. (2023) [23], Liu et al. (2023) [24], and Bastu et al. (2016) [25] were excluded from the mean calculation. * Means calculated from studies that reported standard deviations; data from Sun et al. (2023) [23], Liu et al. (2023) [24], and Bastu et al. (2016) [25] were excluded from these calculations. ** Denotes control group included for comparison; not treated with letrozole. N/A indicates ‘Not Applicable’.

Studies	Letrozole (SD) (n)	Letrozole in Combination (SD) (n)	Other Regimes (SD) (n)	Total Sample (n)
Sun et al., 2023 [23]	11	N/A	10	652
Liu et al., 2023 [24]	N/A	13.5	15	268
Cantor et al., 2019 [26]	N/A	9.1 (2.4)	4 (1.7)	104
Bastu et al., 2016 [25]	N/A	3.45 (1.92)	3.65 (1.50) 3.35 (1.58)	95
Haas et al., 2017 [16]	N/A	14.5 (9.3)	10 (6.0) Control group **	174
Pereira et al., 2017 [27]	N/A	13.1 (7.1) 13.6 (7.5)	14.5 (1.4) 12.5 (7.8)	341
D’Amato et al., 2018 [28]	N/A	6	6	125
Total	11	10.46 *	8.62 *	1759

**Table 5:** Implantation rates by treatment group. Data for each treatment group are displayed as percentages, with the total sample size (n) for each study provided. The mean implantation rates are as follows: natural cycle (57%), letrozole (47.53%), letrozole in combination (34.61%), and other regimens (25.8%). No statistically significant difference was found (*p* = 0.096). The table presents the mean rates from all of the included studies. * Means were calculated based only on studies that reported implantation rates for each respective group; studies with missing or not applicable (N/A) values were excluded from the calculation. N/A indicates ‘Not Applicable’.

Studies	Natural Cycle (%)	Letrozole (%)	Letrozole in Combination (%)	Other Regimens (%)	Total Sample (n)
Sun et al., 2023 [23]	N/A	36.46	N/A	33.92	652
Liu et al., 2023 [24]	N/A	N/A	43.15	38.59	268
Cantor et al., 2019 [26]	N/A	N/A	46	29	104
Bastu et al., 2016 [25]	N/A	N/A	14.7	14.2 13.3	95
Ezoe et al., 2022 [18]	57	58.6	N/A	N/A	3820
Total	57 *	47.53 *	34.61 *	25.8 *	4939

**Table 6:** Clinical pregnancy rates by treatment group. This table summarizes the research findings on clinical pregnancy rates. The overall sample size (n) for each trial is given, and data are shown as percentages for each treatment group. The mean clinical pregnancy rates are as follows: natural cycle (42.4%), letrozole (50.57%), letrozole in combination (41.46%), and other regimens (35.48%). The results showed no statistically significant difference (*p* = 0.42). The table presents mean rates from all included studies. The mean rates were calculated without data from certain research. * Means were calculated based only on studies that reported clinical pregnancy rates for each respective group; studies with missing or not applicable (N/A) values were excluded from the calculation. N/A indicates ‘Not Applicable’.

Studies	Natural Cycle (%)	Letrozole (%)	Letrozole in Combination (%)	Other Regimens (%)	Total Sample (n)
Sun et al., 2023 [23]	N/A	49.43	N/A	47.15	652
Atkinson et al., 2021 [29]	34.4	40.5	N/A	N/A	5395
Liu et al., 2023 [24]	N/A	N/A	53.37	52.85	268
Cantor et al., 2019 [26]	N/A	N/A	50	22.22	104
Bastu et al., 2016 [25]	N/A	N/A	20	22 18	95
Zhang et al., 2021 [30]	N/A	58.4	N/A	54.5	2782
Ezoe et al., 2022 [18]	50.4	54.2	N/A	N/A	3820
L., Wang et al., 2019 [31]	N/A	30	N/A	13.8	160
X., Wang et al., 2023 [19]	N/A	70.9	N/A	64.4	3707
D’Amato et al., 2018 [28]	N/A	N/A	42.5	24.4	125
Total	42.4 *	50.57 *	41.46 *	35.48 *	17,108

**Table 7:** Ongoing pregnancy rates by treatment group. This table summarizes the results of studies reporting the ongoing pregnancy rate. The overall sample size (n) for each trial is given, and the data are shown as percentages for each treatment group. The mean ongoing pregnancy rates are as follows: letrozole (38.29%), letrozole in combination (58.3%), natural cycle (44.5%), and other regimens (25.78%). No statistically significant difference was found (*p* = 0.86). The table presents the mean rates from all included studies. * Means were calculated based only on studies that reported ongoing pregnancy rates for each respective group; studies with missing or not applicable (N/A) values were excluded from the calculation. N/A indicates ‘Not Applicable’.

Studies	Natural Cycle (%)	Letrozole (%)	Letrozole in Combination (%)	Other Regimens (%)	Total Sample (n)
Sun et al., 2023 [23]	N/A	44.19	N/A	41.97	652
Haas et al., 2017 [16]	N/A	N/A	58.3	65.2	174
Ezoe et al., 2022 [18]	44.5	48.2	N/A	N/A	3820
L., Wang et al., 2019 [31]		22.5	N/A	10	160
Total	44.5	38.29 *	58.3	25.98 *	4806

**Table 8:** Miscarriage rates by treatment group. This table provides an overview of the findings from research studies reporting on miscarriage rates. The overall sample size (n) for each trial is given, with data shown as percentages for each treatment group. The mean miscarriage rates are as follows: natural cycle (20.05%), letrozole (14.86%), letrozole in combination (16.89%), and other regimens (16.8%). No statistically significant difference was found (*p* = 0.84). The table presents the mean rates from all included studies. * Means were calculated based only on studies that reported miscarriage rates for each respective group; studies with missing or not applicable (N/A) values were excluded from the calculation. N/A indicates ‘Not Applicable’.

Studies	Natural Cycle (%)	Letrozole (%)	Letrozole in Combination (%)	Other Regimens (%)	Total Sample (n)
Sun et al., 2023 [23]	N/A	5.92	N/A	6.22	652
Atkinson et al., 2021 [29]	17.8	17.6	N/A	N/A	5395
Liu et al., 2023 [24]	N/A	N/A	13.79	15.69	268
Cantor et al., 2019 [26]	N/A	N/A	20	20.3	104
Zhang et al., 2021 [30]	N/A	14.3	N/A	21.7	2782
Ezoe et al., 2022 [18]	22.3	21.8	N/A	N/A	3820
X., Wang et al., 2023 [19]	N/A	14.7	N/A	20.1	3707
Total	20.05 *	14.86 *	16.89 *	16.8 *	16,728

**Table 9:** Live birth rate by treatment groups. This table provides an overview of the studies’ findings on live birth rates. The data for each treatment group are shown as percentages, with the total sample size (n) for each study provided. The mean live birth rates are as follows: natural cycle (33.25%), letrozole (45.58%), letrozole in combination (37.79%), and other regimens (36.98%). No statistically significant difference was found (*p* = 0.5). The table presents the mean rates from all included studies. * Means were calculated based only on studies that reported live birth rates for each respective group; studies with missing or not applicable (N/A) values were excluded from the calculation. N/A indicates ‘Not Applicable’.

Studies	Natural Cycle (%)	Letrozole (%)	Letrozole in Combination (%)	Other Regimens (%)	Total Sample (n)
Sun et al., 2023 [23]	N/A	43.51	N/A	40.93	652
Atkinson et al., 2021 [29]	27.4	32	N/A	N/A	5395
Liu et al., 2023 [24]	N/A	N/A	35.58	34.2	268
Cantor et al., 2019 [26]	N/A	N/A	40	16.67	104
Zhang et al., 2021 [30]	N/A	49.6	N/A	41.7	2782
Ezoe et al., 2022 [18]	39.1	42.3	N/A	N/A	3820
X., Wang et al., 2023 [19]	N/A	60.5	N/A	51.4	3707
Total	33.25 *	45.58 *	37.79 *	36.98 *	16,728

## Data Availability

No new data were created or analyzed in this study.

## Supplementary Materials

The following supporting information can be downloaded at https://www.mdpi.com/article/10.3390/healthcare13131486/s1, Table S1: The PRISMA Checklist for scoping reviews. Reference [38] is cited in Supplementary Materials.
